# The Mechanical Properties, Secondary Structure, and Osteogenic Activity of Photopolymerized Fibroin

**DOI:** 10.3390/polym12030646

**Published:** 2020-03-12

**Authors:** Ivan Bessonov, Anastasia Moysenovich, Anastasia Arkhipova, Mariam Ezernitskaya, Yuri Efremov, Vitaliy Solodilov, Peter Timashev, Konstantin Shaytan, Alexander Shtil, Mikhail Moisenovich

**Affiliations:** 1 Biological Faculty, Lomonosov Moscow State University, 119234 Moscow, Russia; ivanbessonov@gmail.com (I.B.); a-moisenovich@mail.ru (A.M.); anastasia-yu.arkhipova@yandex.ru (A.A.); shaytan49@yandex.ru (K.S.); 2 JSC Efferon, 143026 Moscow, Russia; 3 Regional Research and Clinical Institute (“MONIKI”), 129110 Moscow, Russia; 4 A. N. Nesmeyanov Institute of Organoelement Compounds, Russian Academy of Sciences, 119334 Moscow, Russia; ezernits@mail.ru; 5 Institute for Regenerative Medicine, Sechenov University, 119991 Moscow, Russia; yu.efremov@gmail.com (Y.E.); timashev.peter@gmail.com (P.T.); 6 Semenov Institute of Chemical Physics Russian Academy of Sciences, 119991 Moscow, Russia; vital-yo@yandex.ru; 7 Blokhin National Medical Research Center of Oncology, 115478 Moscow, Russia; shtilaa@yahoo.com; 8 Institute of Gene Biology, Russian Academy of Sciences, 119991 Moscow, Russia

**Keywords:** silk fibroin, methacrylated silk fibroin, tissue engineering, photocrosslinking, osteogenic differentiation

## Abstract

Previously, we have described the preparation of a novel fibroin methacrylamide (FbMA), a polymer network with improved functionality, capable of photocrosslinking into Fb hydrogels with elevated stiffness. However, it was unclear how this new functionality affects the structure of the material and its beta-sheet-associated crystallinity. Here, we show that the proposed method of Fb methacrylation does not disturb the protein’s ability to self-aggregate into the stable beta-sheet-based crystalline domains. Fourier transform infrared spectroscopy (FTIR) shows that, although the precursor ethanol-untreated Fb films exhibited a slightly higher degree of beta-sheet content than the FbMA films (46.9% for Fb-F-aq and 41.5% for FbMA-F-aq), both materials could equally achieve the highest possible beta-sheet content after ethanol treatment (49.8% for Fb-F-et and 49.0% for FbMA-F-et). The elasticity modulus for the FbMA-F-et films was twofold higher than that of the Fb-F-et as measured by the uniaxial tension (130 ± 1 MPa vs. 64 ± 6 MPa), and 1.4 times higher (51 ± 11 MPa vs. 36 ± 4 MPa) as measured by atomic force microscopy. The culturing of human MG63 osteoblast-like cells on Fb-F-et, FbMA-F-et-w/oUV, and FbMA-F-et substrates revealed that the photocrosslinking-induced increment of stiffness increases the area covered by the cells, rearrangement of actin cytoskeleton, and vinculin distribution in focal contacts, altogether enhancing the osteoinductive activity of the substrate.

## 1. Introduction

The mechanical characteristics of scaffolds for tissue engineering are of the utmost importance for cell attachment and differentiation. Cells on the scaffold apply mechanical forces, thereby causing its deformation. In turn, elastic deformation of the substrate provides cytoskeletal tension. Focal contacts initiate the signaling from the surface to the cytoplasm and the nucleus. On the rigid substrate resistant to tension, the cells tend to spread, whereas they are largely round-shaped on soft and easily deformable supports [1,2]. Thus, the substrate’s elasticity modulus (EM) can regulate cell morphology, motility, and the direction of differentiation. The *EM* of normal bone extracellular matrix (ECM) is 20–50 kPa [3], whereas for the mineralized bone, the values are as large as 10–20 GPa [4]. The respective values for typical polymer scaffolds are in the kPa range [5]; an increase in *EM* can be a factor of the osteogenic potency of the material [6,7,8].

Fibroin (Fb) is a perspective source of scaffolds for tissue engineering due to its remarkable biocompatibility, moderate immunomodulating activity, bioresorbability, yield of non-toxic products (mostly amino acids and short peptides), and its mechanical strength [7]. In particular, the fibroin-based materials are well-described scaffolds with perspective osteogenic properties [9,10,11]. In a dry state, the *EM* values of Fb fibers are within the GPa range [12]. However, for porous swollen scaffolds, these parameters normally decrease to 5–25 kPa [13]. This fact can be explained by the specific primary and secondary structure of fibroin [14]. Namely, the repetitive sequences of the hydrophobic amino acid residues assemble into beta sheets that, in turn, form nanoscale crystalline domains within the polymer. Therefore, the Fb-based materials are semicrystalline. The content, localization, and orientation of crystalline domains may depend on the method of material fabrication; therefore, the mechanical properties would be different [15]. Fb modifications at the side groups are promising for synthesis of polymers by an additional functional network [16,17] with optimized characteristics.

Chemically modified Fb substrates have been demonstrated to be promising materials for bone regeneration [17]. In this study, a urethane methacrylate Fb derivative was prepared; photopolymerization of this macromonomer yielded covalently cross-linked films. The degree of cross-linking negatively correlated with *EM* values and beta-sheet crystallinity. The urethane methacrylic Fb derivative [17] has been synthesized using a highly reactive isocyanoethyl methacrylate that provided an exhaustive modification of abundant serine residues (~12% of total amino acid content) in the hydrophobic repeats (GAGAGS) that form the crystalline domains of beta sheets. We have proposed another synthetic route for Fb methacrylation, yielding the methacrylamide derivative FbMA [18]. In doing so, we introduced the methacrylic moieties under mild conditions selectively at the amino groups of Arg and Lys residues, whose total presence in Fb is only ~0.5%. Expectedly, new sparse covalent bonds in the photochemically cross-linked FbMA gel were formed in protein’s amorphous domains. As the amount, size, orientation, and distribution of crystalline domains are essential for superior mechanical characteristics of the material, photocrosslinking via the amorphous domains is beneficial to maintain the remarkable Fb properties. Thus, FbMA, being a convenient parental compound for the photochemical fabrication (including SLA and DLP additive manufacturing) of covalently cross-linked Fb gels, can provide the scaffolds with a higher EM. This property is important for osteogenic potency and makes FbMA preferable over the materials described in [17].

In this study, we report that FbMA-based materials retain the pristine fibroin’s beta-sheet crystallinity and its properties as a scaffold for cell culture. Importantly, the elevated stiffness of FbMA-based materials provides an osteogenic response of human osteoblast-like cells (MG63 line).

## 2. Materials and Methods

### 2.1. Reagents

Methacrylic anhydride (94%), Na_2_CO_3_, LiBr, and diphenyl-(2,4,6-trimethylbenzoyl)phosphine oxide (TPO, 97%) were purchased from Sigma-Aldrich (Darmstadt, Germany). 1,1,1,3,3,3-Hexafluoro-2-propanol (HFIP, 99%) was purchased from P&M–Invest (Moscow, Russia), and ethanol (95%) was purchased from Medchimprom (Moscow Region, Balashikha, Russia).

### 2.2. Isolation of Fb

Lyophilized Fb was obtained from surgical silk threads LLC «Optikum» (Moscow, Russia) using the established protocols [19]. Briefly, threads were boiled for 30 min in aqueous 0.02 M Na_2_CO_3_, and then rinsed for 3 × 30 min in distilled water to remove Na_2_CO_3_. The threads were dried in the oven at 60 °C for 4 h, and then dissolved in 9.3 M aqueous LiBr at 60 °C for 3–4 h. The viscous yellow solution was dialyzed for 2 days in deionized water. The resulting solution was frozen for 2 days, vacuum dried at −20 °C until complete sublimation, and then stored in ambient conditions in closed vials.

### 2.3. Synthesis of FbMA

Methacrylated fibroin (FbMA) was synthesized according to a procedure described by us [18]. Briefly, 1 g of lyophilized Fb was placed in a round-bottom flask equipped with a magnetic stirrer and 20 mL of 0.1 M potassium phosphate buffer solution (pH 7.0) was added. Fb was dissolved on a water bath at 50 °C under continuous stirring to reach the final protein concentration of 5% (*w/v*). One milliliter of methacrylic anhydride was then added to the mixture. The reaction was continued under stirring at 50 °C for 1 h, followed by the addition of 20 mL 0.1 M potassium phosphate buffer solution (pH 7.0) and cooling the reaction mixture to room temperature. The solution was dialyzed against a 20-fold volume of deionized H_2_O through a cellulose cut-off dialysis tubing under constant stirring until the smell of methacrylic anhydride disappeared; water was exchanged with a fresh portion every hour. The resulting product was placed in a Petri dish and frozen at −18 °C. Freeze-drying up to constant mass afforded FbMA as a white powder (yield 97%).

### 2.4. Preparation of Fb-HFIP Solution

Forty milligrams of lyophilized Fb was added to a glass vial with a screw cap and equipped with a magnetic stirrer. Four milliliters of HFIP was added to the vial. Fibroin and HFIP were kept in a closed vial under a continuous stirring for 2 h at 50 °C, until a clear yellow viscous solution was obtained. The solution was stored at ambient temperature before the films were cast.

### 2.5. Preparation of FbMA-HFIP Solution

Forty milligrams of powdered FbMA were added to an amber glass vial with a screw cap and equipped with magnetic stirrer. Two milligrams of TPO photoinitiator and 4 mL of HFIP were added, and the vials were kept in the dark. FbMA, TPO, and HFIP were kept in the closed vial under continuous stirring for 2 h at 50 °C until a clear yellow viscous solution was obtained. The prepared solution was stored at ambient temperature before the films were cast.

### 2.6. Film Fabrication

Six-hundred microliters of 10 mg/mL Fb/HFIP solution was pipetted on a smooth surface of an open-type mold (flat injection-molded polypropylene sheet) and left for 2 h at room temperature under the fume hood. As the solvent evaporated, a transparent film was detached from the mold and placed into 50 mL of sterile 0.9% aqueous NaCl. The solution was changed every 8 h for 48 h. The washed film was stored in a tightly closed vessel under sterile 0.9% NaCl solution at 4 °C.

The Fb-F-aq film was placed into 70% aqueous ethanol for 2 h, and then into 96% ethanol for 24 h. The dehydrated films were kept under 96% ethanol in a tightly closed vessel at 4 °C.

To obtain FbMA-F-aq, 600 µL of 10 mg/mL FbMA/HFIP solution was pipetted on a smooth surface of an open-type mold (flat injection-molded polypropylene sheet) and left for 2 h at room temperature under the fume hood. The dry film was formed as described above. Then, the film was irradiated with 365 nm UV light at an intensity of 10 mW/cm^2^ for 10 min and placed into 50 mL of sterile 0.9% aqueous NaCl. The solution was changed every 8 h for 48 h. The washed film was stored in a tightly closed vessel under sterile 0.9% NaCl solution at 4 °C.

The FbMA-F-aq film was placed into 70% aqueous ethanol for 2 h, and then into 96% ethanol for 24 h. The dehydrated films were kept under 96% ethanol in a tightly closed vessel at 4 °C.

FbMA-F-et-w/oUV were obtained as described above, but the UV irradiation step was omitted.

### 2.7. Fourier Transform Infrared Spectroscopy (FTIR)

Fourier transform infrared spectroscopy (FTIR) spectra were obtained on a Vertex 70 V Fourier spectrometer (Bruker, Ettlingen, Germany) using an ATR accessory with a diamond crystal (Pike, Fitchburg, WI, USA); the ATR spectra were collected in vacuum after 128,256 scans over a range of 4000 to 400 cm^−1^ with a resolution of 4 cm^−1^. All corrections were done using an Omnic 8 program package.

### 2.8. Mechanical Tests

To determine the mechanical characteristics of studied materials, T-bone-shaped test specimens were obtained from the films by applying the cutting method. A series of 5 test specimens were cut for each type of material. Sample dimensions: thickness of 40 µm, width of 6 mm, and bearing length of 33 mm. One day prior to testing, Fb-F-et and FbMA-F-et samples were transferred from ethanol solution into an excessive volume of 0.9% NaCl.

The thin paper soaked in 0.9% NaCl was stuck to the surface to prevent drying during the test. As soaking disrupts the paper to individual fibers, its effect on mechanical characteristics of the films was negligible. Uniaxial tension was performed on a Zwick Z 100 test machine at a load rate 10 mm/min. Stress–strain curves (*σ–ε*) were registered during the loading. These curves were used to determine (according to ASTM D882-12) conditional yield strength, *σ_T_*; at conditional elongation, *ε_T_*; tensile strength, *σ_p_*; elastic modulus, *E*, and elongation at break, *ε_P_*. Standard deviations were calculated for each value.

### 2.9. Atomic Force Microscopy (AFM)

Nanomechanical measurements were performed on a Bioscope Resolve Atomic Force Microscopy (AFM) (Bruker, Santa Barbara, CA, USA) system mounted on an Axio Observer inverted optical microscope (Carl Zeiss, Jena, Germany). The ScanAsyst-Fluid cantilevers were used with the nominal spring constant 0.7 N/m and the nominal tip radius 20 nm. The exact values were determined by thermal tune method and by scanning of the titanium roughness sample (Bruker), respectively. All AFM measurements were performed in phosphate-buffered saline at room temperature. Force volume mapping was performed over 10 × 10 µm areas with 16 × 16 force curve arrays. Three samples of each film were analyzed (3–6 random areas per sample). The vertical piezo movement speed was 2 µm/s, and the trigger force was ≈25 nN. Each force curve was analyzed to extract the Young’s modulus value using the NanoScope Analysis software (Bruker) with the Hertz model [20]:

$$F=\frac{4}{3}\frac{E}{1-v^{2}}\sqrt{R}\delta^{\frac{3}{2}}$$

where *F* is the measured force, *E* is the Young’s modulus, *ν* is the Poisson ratio (assumed to be 0.5), *R* is the tip radius, and *δ* is the indentation depth.

### 2.10. Culturing Human MG63 Osteoblast-Like Cells on Fb Substrates

The Fb-F-et, FbMA-F-et-w/oUV, and FbMA-F-et films were sterilized in 70% ethanol overnight, washed thrice with Eagle-MEM containing 1% nonessential amino acids (NEAA), then five times with Eagle-MEM supplemented with 10% fetal bovine serum (FBS) and 1% NEAA (30 min each washing) at 37 °C, 5% CO_2_, transferred to Petri dishes with fresh medium and left overnight at 37 °C, 5% CO_2_. The medium was discarded, 2 mL of MG63 (ATCC^®^ CRL1427™) cell suspension (2.3 × 10^4^ cells) in Eagle-MEM supplemented with 10% FBS and 1% NEAA. Then, cells were incubated overnight at 37 °C, 5% CO_2_. By day 7, in culture the medium was changed for an osteogenic medium containing α-MEM with 5% FBS, 1% NEAA, 1% β-glycerophosphate, 0.01% dexamethasone, and 0.1% ascorbic acid, and left in the incubator for 14 days. The medium was changed every 3 days.

### 2.11. Cell Viability Tests

By days 1, 4, and 7 of culturing MG63 cells on Fb-F-et, FbMA-F-et-w/oUV, and FbMA-F-et surface cell viability was assessed by reducing 3-(4,5-dimethylthiazol-2-yl)-2,5-diphenyltetrazolium bromide (MTT tests). Films with cells were transferred into Petri dishes containing 2 mL of serum-free Eagle-MEM and 625 µg/mL MTT reagent, and incubated for 4 h at 37 °C, 5% CO_2_. Then films were placed in DMSO to dissolve formazan, centrifuged at 16,800 g for 5 min. The supernatant was transferred into a 96-well plate. Optical density was measured at 550 nm. Films without cells were similarly processed as a control.

### 2.12. Cytoskeleton Morphology

By day 1 of culturing on Fb-F-et, FbMA-F-et-w/oUV and FbMA-F-et the MG63 cells were fixed with 4% paraformaldehyde in saline for 30 min at room temperature in the dark followed by three washings in saline. Then, the cells were permeabilized with 0.1% Triton X-100 in saline supplemented with 0.1% FBS for 10 min at 4 °C and washed twice with saline/0.1% FBS. Non-specific protein binding was blocked with PBS/1% FBS/0.1% Tween-20. To visualize focal contacts, cells were treated with mouse anti-vinculin antibody (ThermoFisher Scientific, Waltham, MA, USA, 1:200 in PBS/0/1% FBS/0/1% Tween-20; 1 h). After washing the secondary CF™ 543, conjugated anti-mouse IgG (H + L) (Sigma-Aldrich; Darmstadt, Germany; 1:700) was added. To visualize the actin cytoskeleton, cells were incubated with phalloidin conjugated with Alexa488 (Sigma-Aldrich, Darmstadt, Germany) as recommended by the manufacturer, and washed three times with saline. Hoechst 33342 ((ThermoFisher Scientific™, Waltham, MA, USA; 1 µg/mL) was added to counterstain the nuclei. Samples were analyzed on a Nikon Ti-E microscope with a confocal module A1 and the objective Apo TIRF Plan Fluor 63 × 1.49.

### 2.13. Activity of Alkaline Phosphatase (ALP)

The activity of Alkaline Phosphatase (ALP) was determined at days 7 and 14 of culture of MG63 cells on Fb-F-et, FbMA-F-et-w/oUV, and FbMA-F-et films in the osteogenic medium. Cells on the films were lysed in the buffer (50 mM Tris base, 100 mM glycin and 0.1% Triton X-100, XH 10.5). One milliliter of colorless p-nitrophenylphosphate (10 mg/mL), an ALP substrate, was added. Lysates were placed in the dark and incubated for 30 min. The reactions were stopped with 0.5 mL 0.2 M NaOH. ALP hydrolyzes p-nitrophenylphosphate to form a yellow p-nitrophenol. The activity of ALP was assessed by optical density at 405 nm. Values of ALP activity were normalized to the values in MTT tests. Films without cells were used as a control.

### 2.14. Deposition of Calcium Phosphate by MG63 Cells on Fb Scaffolds

By day 14 of culturing MG63 cells on Fb-F-et, FbMA-F-et-w/oUV, and FbMA-F-et under osteogenic conditions, samples were fixed in 2.5% glutaraldehyde, washed three times with deionized water, placed into 1.5 mL of 2% solution of alizarin red (pH 4.1–4.3), and incubated in the dark for 1 h at room temperature. Then, samples were washed 5 times with water. Calcium salts were examined on an inverted microscope Axiovert 200 M, Plan-Neofluar 20×/0.5 objective and AxioCam MRC 5 camera (Carl Zeiss, Jena, Germany). For quantitative analysis, samples stained with alizarin red were put into 1 mL of 10% CPC solution for 1 h. The optical density of the supernatant was measured at 540 nm and normalized by MTT values. Films without cells were used for control.

## 3. Results

### 3.1. Film Fabrication

**Figure 1:**
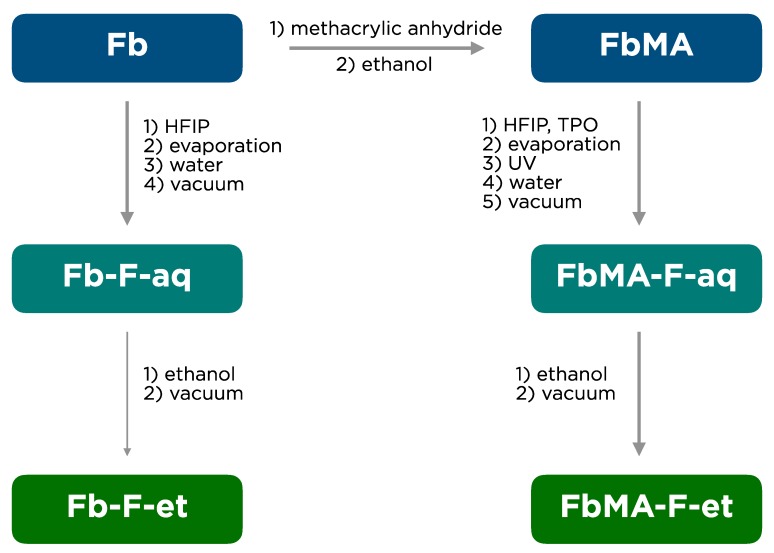
Preparation of fibroin (Fb) films.

The experimental scheme of fibroin films preparation is shown in Figure 1.

Pristine Fb as a lyophilisate was used for synthesis of its methacrylated derivative FbMA [18]. To fabricate the films, these proteins were dissolved in HFIP. The Fb-F-aq film was fabricated from pristine Fb solution in HFIP by solvent evaporation and subsequent treatment with water. The FbMA-F-aq film was fabricated in a similar manner, but before its transfer into water, the film was subjected to UV irradiation in the presence of photoinitiator TPO to form covalent carbon–carbon bonds by the methacrylic residues. FbMA-F-et and Fb-F-et films were fabricated upon treatment of the corresponding water-treated precursor films with an excess of ethanol.

### 3.2. Mechanical Properties

**Figure 2:**
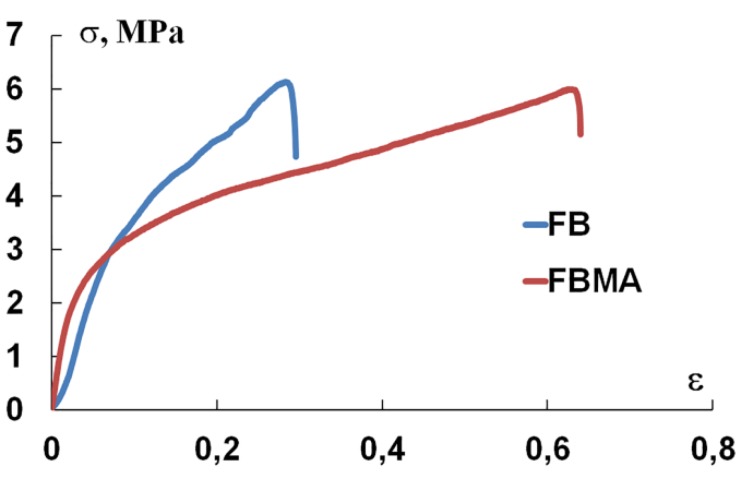
Stress–strain curve of Fb-F-et and FbMA-F-et films.

Figure 2 shows that both Fb-F-et and FbMA-F-et were deformed elastically, i.e., the stress arisen in the material was directly proportional to elongation. Notably, the linear sections of the diagrams do not coincide. However, the curve of Fb-F-et is steeper. Further loading led to irreversible deformation. The bend of the loading diagram corresponds to the material’s yield strength, which is the same for both materials.

Table 1 summarizes the mechanical characteristics of the films. The conditional yield strength *σ_T_* is lower than the tensile strength *σ_p_*, although these values are within the same range. This indicates that, during the tension process, the films become more stiff. The orientation of crystalline domains in an amorphous matrix further increases the strength of the films. *EM* values differed twofold: 64 MPa for Fb-F-et compared to 130 MPa for FbMA-F-et. The yield strength for FbMA-F-et was achievable at 4% extension vs. 9% for Fb-F-et.

**Figure 3:**
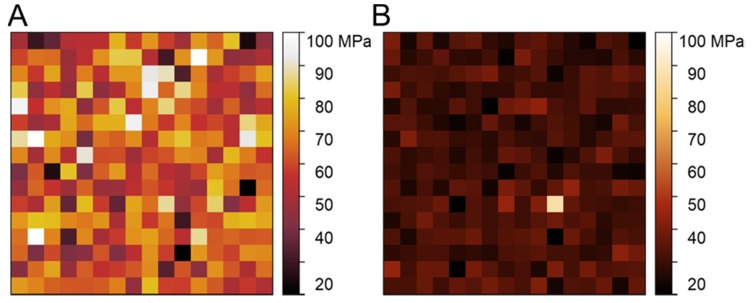
Nanomechanical maps (Young’s modulus distributions) over a 10 × 10 µm area mapped using the Atomic Force Microscopy (AFM) Force Volume mode. (**A**) Representative map over the FbMA-F-et film. (**B**) Representative map over the Fb-F-et film. The color-coded scale of the Young’s modulus is the same for both maps to better represent the difference. Note a significantly larger and more heterogeneous Young’s modulus of FbMA-F-et film compared to Fb-F-et film.

Also, the mechanical properties of the films were assessed by AFM nanoindentation experiments. The films were attached to the surface. Unlike uniaxial tension, which provides information about mechanics on a macro-scale (bulk properties), AFM analyses the properties specifically in the surface layer on a 10–100 nm scale. This scale is especially relevant considering the size of the structures responsible for the cell–surface interaction (focal adhesions), that is, hundreds of nm to several µm [21]. Examples of AFM nanomechanical maps are shown in Figure 3. Overall, the FbMA-F-et film showed a significantly higher (51 ± 11 MPa), but also more heterogeneous, Young’s modulus than Fb-F-et film (36 ± 4 MPa).

### 3.3. Secondary Structure

**Figure 4:**
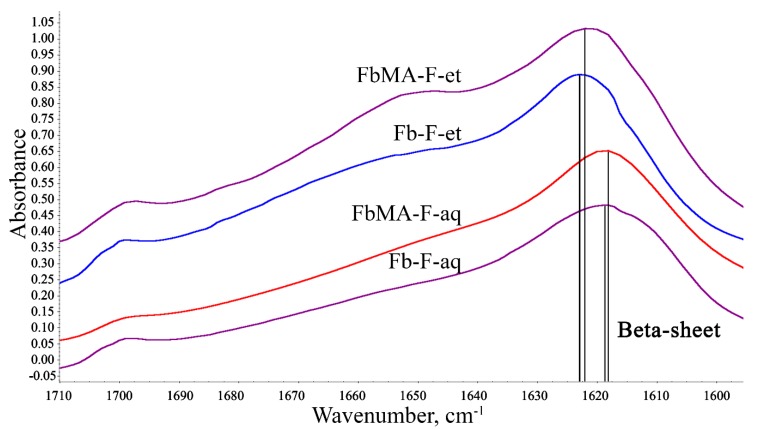
IR spectra of Fb-F-aq, FbMA-F-aq, Fb-F-et, and FbMA-F-et. Double lines show main peaks of beta sheets.

FTIR spectroscopy was used to evaluate the degree of beta-sheet content (which correlates with crystallinity) of the materials. The shape of Amid I broad band (1590–1710 cm^−1^) in the IR spectra of Fb provides information about the secondary structure of the protein [22]. Figure 4 depicts the Amid I spectral range for all four films.

**Figure 5:**
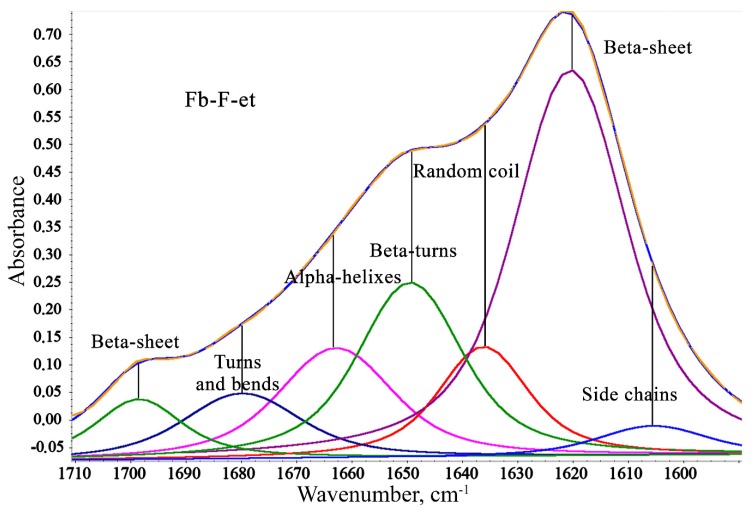
Individual bands that comprise the Amid I band in IR spectrum of Fb-F-et. Two bands at 1610–1635 cm^−1^ and 1695–1710 cm^−1^ correspond to the beta sheet structures.

A broad Amid I band at ~1600–1700 cm^−1^ summarizes the individual stretching modes of amide groups of the secondary structures within the protein [23,24]. This band was resolved into individual components whose integral intensities were normalized to total intensity of the Amid I band. As the degree of beta-sheet content is a key determinant of Fb mechanical properties, the components in the ranges 1610 to 1635 cm^−1^ and 1695 to 1710 cm^−1^ assigned to β-sheets were the most important for interpretation. A general view of the respective spectral region with individual bands that correspond to the secondary structures of Fb-F-et is shown in Figure 5. Integral intensities of the peaks were used for quantitation of the impact of each structure (Table 2).

Table 2 presents data for Fb-F-et and FbMA-F-et and their precursors Fb-F-aq and FbMA-F-aq, respectively. For Fb-F-aq, the total content of beta sheets was 46.9%, whereas for FbMA-F-aq, this value was slightly lower (41.5%). Other types of secondary structures also differed between the materials. The content of the side chains, alpha-helixes, bends, and turns was greater for FbMA-F-aq, whereas random coils and beta-turns were more frequent in Fb-F-aq films. However, Fb-F-et and FbMA-F-et treated with ethanol had similar percentages of beta-sheets: 49.0% and 49.8%, respectively. These two materials possess a maximal degree of beta-sheet content. No substantial differences between other types of secondary structures were observed after treatment with ethanol.

### 3.4. Biological Properties

**Figure 6:**
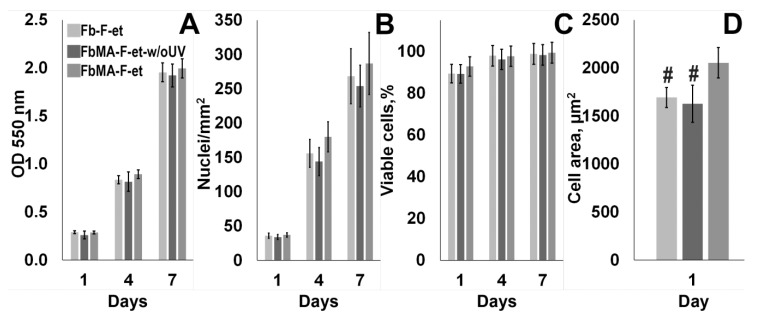
Cytocompatibility of FbMA-F-et. (**A**) Cell survival (MTT tests) at days 1, 4, and 7 of culture; (**B**) Number of nuclei per 1 mm^2^; (**C**) % viable cells by LIVE/DEAD tests. (**D**) Cell area at one day of cultivation. #: statistically significant difference with FbMA-F-et group (*p* < 0.05).

Films made of Fb-F-et and FbMA-F-et were used for culturing the MG63 cells. As shown in Figure 6A–C, no statistically significant changes in cell number, survival, and spreading were detectable between the cells grown on each substrate (*p* > 0.05).

**Figure 7:**
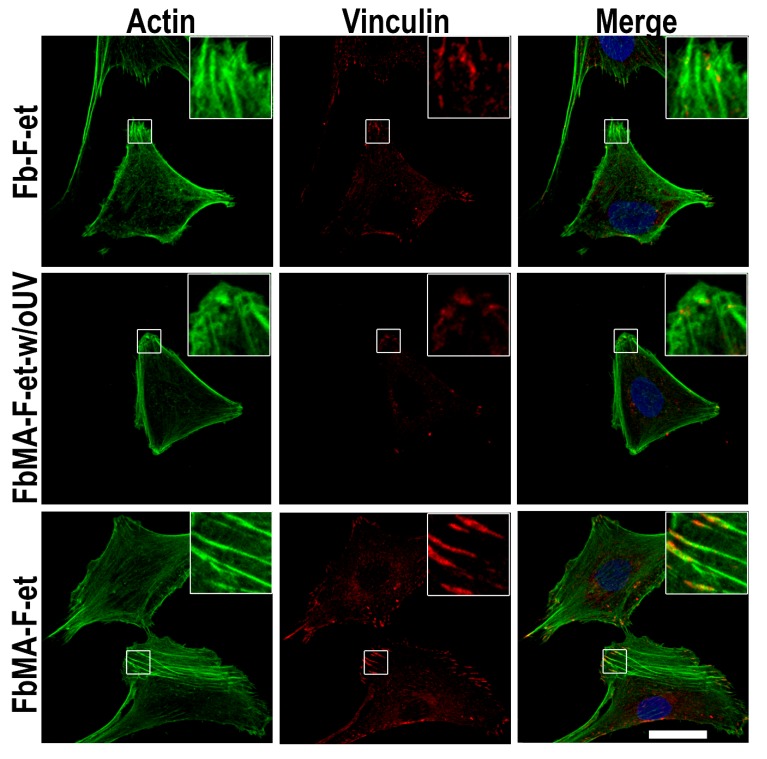
Stress fibers and vinculin organization of MG63 cells after 24 h on Fb-F-et, FbMA-F-et-w/oUV, or FbMA-F-et. Reconstructions are based on optical sections of cells growing on Fb-F-et (top), FbMA-F-et-w/oUV (middle), and FbMA-F-et (bottom). The cytoskeletal network was detected with Alexa488-phalloidin (green), and nuclei were stained with Hoechst 33,342 (blue). Vinculin (red) was visualized by immunocytochemistry. Scale bar, 25 µm.

A comparison of focal contacts and actin microfilaments in MG63 cells revealed that on Fb-F-et and FbMA-F-et-w/oUV F-actin was less ordered, and the number of stress fibers was smaller than on FbMA-F-et. FbMA-F-et films increased the cell surface compared to Fb-F-et and FbMA-F-et-w/oUV: 2003.75 ± 157.35 µm^2^ vs. 1692.35 ± 103.74 µm^2^ and 1627.15 ± 193.54, respectively (Figure 6D, *p* < 0.05). Furthermore, in cells grown on FbMA-F-et, vinculin was detectable in the cytoplasm, in focal contacts, as well as in the perinuclear area. In the cells cultured on Fb-F-et and FbMA-F-et-w/oUV, the amount of vinculin in the cytoplasm was rather small, and its structure in the focal contacts was less organized (Figure 7).

**Figure 8:**
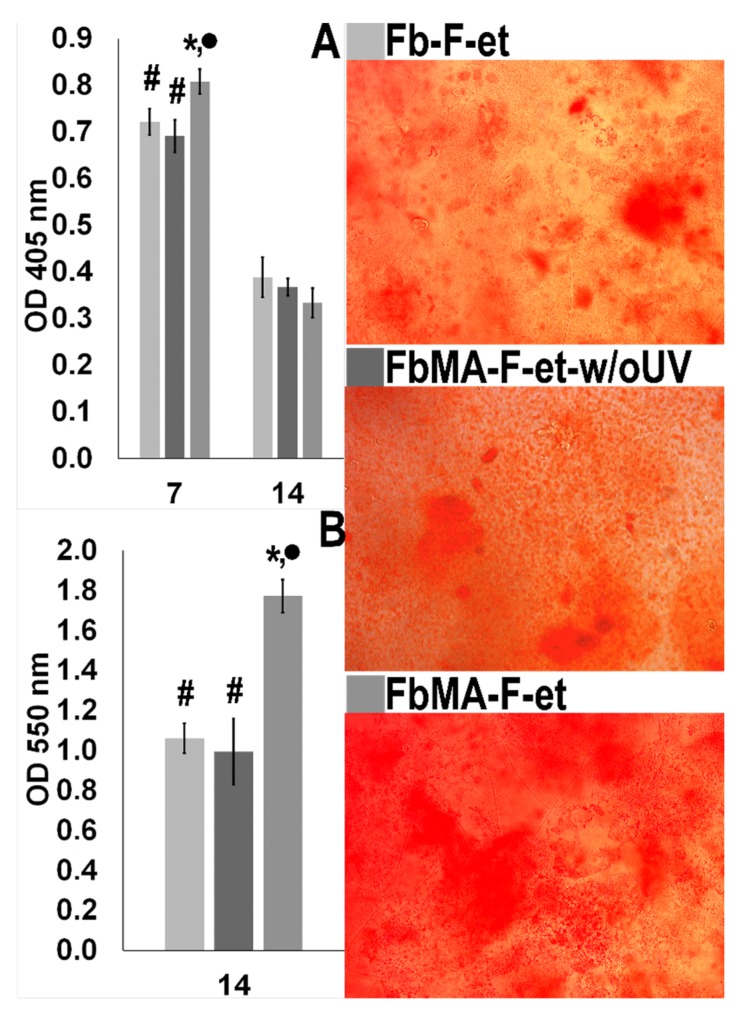
Osteogenic properties of Fb-derived substrates. (**A**) ALP activity by days 7 and 14 after induction of osteogenesis. (**B**) Quantitation of alizarin red staining after 14 days. Right panel: mineral depositions after 14 days of culturing MG63 cells under osteogenic conditions (staining with alizarin red). Statistically significant difference with * Fb-F-et group (*p* < 0.05); • FbMA-F-et-w/oUV (*p* < 0.05); # FbMA-F-et (*p* < 0.05).

Next, we determined ALP (an early marker of osteogenic activity of osteoblasts) and mineralization of Fb-F-et, FbMA-F-et and FbMA-F-et-w/oUV surfaces by MG63 cells. Maximum ALP activity was detectable by day 7; FbMA-F-et was more potent than Fb-F-et and FbMA-F-et-w/oUV (0.808 ± 0.027 vs. 0.721 ± 0.028 and 0.691 ± 0.047), respectively; *p* < 0.05). By day 14, ALP activity decreased in all groups (0.334 ± 0.031 vs. 0.388 ± 0.043 and 0.367 ± 0.037, respectively; *p* > 0.05; Figure 8A). At day 14, alizarin red staining in all groups was positive, indicating the deposition of calcium (Figure 8 right panel). On FbMA-F-et, calcium deposition was more pronounced compared to Fb-F-et and FbMA-F-et-w/oUV (OD values 1.772 ± 0.081 vs. 1.061 ± 0.074 and 0.994 ± 0.17, respectively; *p* < 0,05) (Figure 8B).

## 4. Discussion

### 4.1. Film Fabrication

In this study, we compared the structure and characteristics of two types of 2D fibroin films (Figure 1). Fibron films are an important type of scaffolds for soft [25] and bone tissue regeneration [26]. The control samples are Fb-F-aq and Fb-F-et films based on the pristine Fb. These materials were fabricated by standard methods (dissolution in HFIP, solution casting, solvent evaporation, and optional ethanol treatment). HFIP, a highly efficient organic solvent for fibroin solubilization, has been used for fabrication of advanced protein-based materials [27]. FbMA-F-aq and FbMA-F-et films were fabricated via TPO-initiated photochemical polymerization of the novel methacrylated Fb derivative FbMA. TPO is a monoacylphosphine-type, highly efficient photoinitiator that undergoes homolytic C–P bond cleavage under UV irradiation [28]. In situ generated free radicals initiate chain polymerization of methacrylic moieties of FbMA, that is, the solid cast FbMA film. Usually, Fb-based materials fabricated without a dehydrating step, such as alcohol treatment, are amorphous; their *EM* values are low («silk I»). Treatment with methanol or ethanol induced protein transition to a crystalline state and provided the material with a higher *EM* value («silk II»). This method of crystallization is critical for making rigid Fb scaffolds for bone regeneration. FbMA-F-aq specimens, unlike Fb-F-aq, possessed an additional network of covalent bonds that limit the mobility of polymer chains. The roles of methacrylic substituents and the cross-links in FbMA-based materials are important for the completion of crystallization and might even negatively influence this process [17]. This factor could alter their conformational transitions to beta-sheets, thereby preventing the formation of the crystalline structure in FbMA-F-et films. On the contrary, similar beta-sheet content for both ethanol-treated samples (Fb-F-et and FbMA-F-et) would indicate that the protein’s self-assembly into a crystalline structure remained unaltered.

### 4.2. Mechanical Properties

The most dramatic difference between FbMA-F-et and Fb-F-et is *EM*: 130 ± 1 MPa vs. 64 ± 6 MPa, respectively (Table 1). Cross-linked FbMA-F-et film also showed a twofold lower elongation (0.4 vs. 0.9) (Table 1). We attributed this differential elasticity to the structure of new polymers (Figure 2). Additional covalent cross-links in FbMA-F-et limit the mobility of peptide chains in the swollen polymer. These findings differ from structurally similar materials reported elsewhere [17]. Using dry specimens, the authors have shown that *EM* decreased from 1.5 GPa for the non-cross-linked Fb to 0.5 GPa for the cross-linked counterpart. Thus, our method of Fb functionalization has no negative influence on mechanical characteristics. Therefore, photochemical cross-linking is relevant for scaffold preparation, so as this process does not decrease the rigidity of the material.

The higher *EM* values of FbMA-F-et film were confirmed by AFM: the film was ~40% stiffer than the Fb-F-et film (51 ± 11 MPa vs. 36 ± 4 MPa, respectively) (Figure 3). These values, however, were lower than those measured with the uniaxial tension. Such discrepancy is observed when macroscale and microscale measurements on the polymer samples are compared; one explanation is a difference between the properties of bulk and surface layers [29]. The higher local heterogeneity of FbMA-F-et films (Figure 3A) could be due to distribution of additional covalent cross-links [30]. Thus, covalent cross-linking of FbMA-F-et did not alter the strength and also increased the *EM* values. This elevated rigidity makes FbMA-F-et similar to the osseous tissue, further implicating this material for bone repair.

### 4.3. Secondary Structure

Conversion of silk I into silk II and quantification of crystallinity can be monitored by FTIR spectroscopy (Figure 4 and Figure 5) [31,32]. HFIP-based Fb-F-aq and FbMA-F-aq films prepared without ethanol treatment exhibit lowered beta-sheet content on comparison to their ethanol-treated counterparts Fb-F-et and FbMA-F-et (Table 2). A beta-sheet content of 46.9% for Fb-F-aq is in an agreement with previously reported 48% for HFIP-based porous fibroin scaffolds [33]. These values are within the range of beta-sheet content achievable with alcohol treatment or similar dehydration step. These results indicate that the Fb-F-aq film is prone to spontaneous crystallization (prompted by vacuum at ATR accessory and the contact with residual NaCl from saline solution).

Chemically modified FbMA-F-aq, indeed, exhibits lowered beta-sheet content—41.5% (Table 2). In contrast to pristine fibroin, the covalently cross-linked methacrylic groups in FbMA-F-aq provide steric hindrance for conformational transition.

Treatment of these films with ethanol yields Fb-F-et and FbMA-F-et films. Despite the structural differences caused by covalent cross-links at the methacrylic substituents in the side chains, Fb-F-et and FbMA-F-et specimens showed similar degrees of beta-sheet content (49.0% vs. 49.8%; Table 2), as determined by FTIR spectra. Neither the introduction of methacrylic groups nor the novel –C–C– bond formation has deteriorated beta-sheet formation. We attributed this result to the repetitive Ala/Gly sequences that are not targeted by methacrylation; these sequences are found in Fb domains critical for beta-sheet formation, so the Fb-to-FbMA transition is not associated with the changes of amino acid residues responsible for assembly into a secondary structure. In contrast, the hydrophilic domains enriched in Lys and Arg residues (into which the methacrylic groups were introduced) do not participate in the formation of the crystalline structure. One may suggest that the observed ~50% degree of beta-sheet content is maximal for «silk II»-type structure prepared by ethanol treatment. This value corresponds to the reported 51.2% beta-sheet content of Fb screws for orthopedic applications fabricated using intermediate HFIP solution and subsequent alcohol treatment [34]. Therefore, the observed differences in mechanical parameters such as EM, deformation, water contact angle, and swelling, are most likely due to the new covalent bonding rather than differential secondary structure. These results disagree with previous reports [17] in which the degree of beta-sheet content of photopolymerized Fb derivatives decreased along with the increased degree of cross-linking. We tend to explain this discrepancy by a specific composition of FbMA polymer. Indeed, in this material, the methacrylic residues were introduced into the amorphous domains, whereas in [17], the authors modified the serine residues involved in the formation of crystalline domains. As expected, direct methacrylation of serine residues prevented self-assembly of crystalline domains, a process dependent on GAGAGS repeats.

Consequently, crystallization of FbMA-F-aq in mild conditions (ambient temperature and low pressure at ATR accessory) is achievable with a lesser conversion than ethanol-induced crystallization. Harsh conditions, such as the prolonged treatment with excess of ethanol, provide the specimens with the same beta sheet content (Fb-F-et and FbMA-F-et). In other words, the covalently cross-linked FbMA-F-aq is less prone to spontaneous beta sheet formation compared to pristine Fb-F-aq. Nevertheless, both Fb-F-et and FbMA-F-et can achieve similar degrees of beta-sheet content at the thermodynamically equilibrium state.

### 4.4. Biological Properties

Materials made of native Fb are known to be highly biocompatible; moreover, these materials are applicable for bone regeneration [7,35,36] and fracture fixation [34,37]. These factors are associated with large mechanical strength and the ability to imitate the anionic charge of non-collagen proteins in the bone ECM, as well as with nucleation of hydroxyapatite and integrin mediated cell adhesion [38]. However, the rigidity of native silk is smaller compared to that in [4], so introduction of methacrylic moieties makes the material similar to the osseous tissue. Our study showed that the MG63 cells can attach, proliferate, and survive on FbMA-F-et films, indicating a good cytocompatibility of the scaffold (Figure 6A–C) [18,39]. Cell–substrate interactions are crucial for a plethora of physiological processes, including cell growth and differentiation, tissue remodeling, regeneration, viability, immune responses, etc. [40,41]. Physical parameters of the solid substrate important for the above interactions are topography, hydrophilicity, and rigidity [42]. As the geometry and the roughness of our materials were similar, differences in cell behavior can be attributable to the viscosity, rigidity, and hydrophilicity of the supports.

The increased cell area has been considered as a hallmark of osteogenic differentiation. Substrates that promote cell spreading are known to enhance osteogenesis, whereas the scaffolds on which the spreading is limited to retard this process [43]. The increased cell spreading is associated largely with dynamic polymerization of actin, that is, the formation of filamentous forms and subsequent restructuring into oriented stress fibers. Importantly, inhibition of actin polymerization attenuates the osteogenic differentiation [44]. We found that the area covered by the cells on the more rigid FbMA-F-et substrate was larger, with longer actin stress fibers along the cell bodies (Figure 6D and Figure 7). In contrast, in the cells cultured on the softer Fb-F-et, actin was diffuse and amorphous although the stress fibers were visible across the cell body (Figure 7). Consequently, the markers of osteogenic differentiation such as ALP and calcium deposition were more pronounced on FbMA-F-et than on Fb-F-et. FbMA-F-et-w/oUV exerted the same effect as Fb-F-et.

Vinculin, a major component of focal contacts, is important for mechanosensing and signal transduction. Deregulation of vinculin is critical for cell adhesion, motility, and proliferation [45]. In particular, vinculin binds actin, thereby stimulating its polymerization and attracting the actin remodeling proteins [46]. Fb-F-et and FbMA-F-et evoked differential effects on morphology of MG63 cells. FbMA-F-et substrate provided formation of mature adhesion contacts at the periphery. Vinculin distribution on Fb-F-et was less organized than on FbMA-F-et (Figure 7). These results corroborate other findings suggesting that the cells adjust their shape and cytoskeleton to the stiffness of the substrate [47]. Interestingly, ALP activity and matrix mineralization were substantially more pronounced on FbMA-F-et (Figure 8). Therefore, in agreement with other studies [48], morphological changes are paralleled by biochemical markers of osteogenesis.

Introduction of methacrylic groups into the FbMA macromonomer occurs at the polar charged amino groups of Lys and Arg residues. The surface of FbMA-F-et is less polar, as the water contact angle is smaller (71° for FbMA-F-et vs. 60° for Fb-F-et) [18]. Furthermore, neutralization of the positive charge in the amino groups by methacrylation is expected to attenuate the net electric charge of the polymer. The roles of the charge and hydrophobicity of the substrate in ECM mineralization and ALP activity, as well as vinculin distribution in osteoblastic MC3T3-E1 cells on self-assembled monolayers of alkanethiols on gold with surface functionalization by –CH3, –OH, –COOH, and –NH_2_ moieties, have been addressed in [49,50]. Cells cultured on hydrophilic surfaces showed elevated ALP activity and ECM mineralization. Also, genes specific for osteogenesis were upregulated compared to the cells cultured on the substrates functionalized by alkyl and carboxylic groups [36]. Both negative and positive charges promote the recruitment of vinculin during focal adhesion assembly in comparison with neutral and hydrophobic surfaces. Moreover, vinculin clusterization positively correlated with the presence of polar groups in the substrate [49]. One might expect a decreased vinculin in focal contacts and an attenuated ALP activity and mineralization on more hydrophobic substrates; however, we did not observe these phenomena. One explanation is that the effects of the increased rigidity prevailed over the effects of the lowered charge and surface hydrophilicity. On the other hand, hydrophobicity and the lowered charge can be compensated by the ability of Fb to adsorb positively charged hydrophilic molecules in the plasma, e.g., fibronectin and laminin, thereby altering the physico-chemical properties of the surface and countering the effects of hydrophobicity and negative charge on cellular functions [51].

## 5. Conclusions

Silk fibroin is one of the most prominent biomaterials for tissue regeneration; its complicated processability into the scaffolds of complex shape might be a major hindrance for its clinical applications. Previously, we have described a new mild method of Fb functionalization of its hydrophilic amorphous domains into the photocrosslinkable macromonomer FbMA. This approach to augment fibroin functionality accommodates it to SLA and DLP additive manufacturing technologies. This study provides further mechanical, structural, and biological evaluation of fabricated materials. Our findings indicate that the proposed method of Fb transformation yields the materials with a twice higher elasticity modulus, keeping the protein’s beta-sheet crystallinity unaltered. FbMA-based materials retain the remarkable properties of Fb as a cell culture substrate, while enhancing their intrinsic osteoinductive properties via mechanical cues.

## Figures and Tables

**Table 1:** Mechanical parameters of Fb-F-et and FbMA-F-et films.

	Parameter	Conditional Yield Strength *σ_T_*, MPa	Tensile Strength *σ_p_*, MPa	*EM*, MPa	Conditional Elongation, *ε_T_* at *σ_T_*	Elongation, *ε_p_* at *σ_p_*
Sample						
FbMA-F-et		2.8 ± 0.5	5.8 ± 0.5	130 ± 1	0.4 ± 0.1	0.61 ± 0.12
Fb-F-et		3.5 ± 0.4	6.9 ± 0.8	64 ± 6	0.9 ± 0.2	0.43 ± 0.14

**Table 2:** FTIR spectra and secondary structures of aqueous Fb and FbMA films.

Secondary Structure	Wavenumber, cm^−1^	FbMA-F-aq	Fb-F-aq	FbMA-F-et	Fb-F-et
Side chains	1590–1605	10.8	6.5	2.8	3.2
Beta-sheets	1610–1635, 1695–1710	41.5	46.9	49.8	49.0
Random coils	1635–1645	10.8	16.0	9.8	9.9
Beta-turns	1647–1654	12.3	15.1	18.2	18.1
Alpha-helixes	1658–1664	13.8	9.4	11.9	12.3
Bends and turns	1666–1695	10.8	6.2	7.4	7.5
